# The role of *Clostridium butyricum* and its metabolites in modulating gut mucosal immunity: implications for viral infections and inflammatory diseases

**DOI:** 10.3389/fimmu.2026.1763817

**Published:** 2026-02-18

**Authors:** Shaoju Qian, Siyu Li, Keyan Ye, Shuao Lu, Xiaoming Sha, Danqiong Zhang, Zhishan Xu, Xiangfeng Song, Ruixue Li

**Affiliations:** 1 School of Basic Medical Sciences, Xinxiang Medical University, Xinxiang, China; 2 Xinxiang Key Laboratory of Tumor Vaccine and Immunotherapy, School of Basic Medical Sciences, Xinxiang Medical University, Xinxiang, China; 3 Xinxiang Engineering Technology Research Center of Immune Checkpoint Drug for Liver-Intestinal Tumors, Xinxiang Medical University, Xinxiang, Henan, China; 4 Department of Otolaryngology, The First Affiliated Hospital of Xinxiang Medical University, Xinxiang, China

**Keywords:** antiviral effects, *Clostridium butyricum*, intestinal barrier, mucosal immunity, relieve inflammation, short-chain fatty acids

## Abstract

With global viral emergence and increasing antiviral resistance, there is an urgent need for innovative immunomodulatory strategies. Gut microbiota modulation has gained attention as a promising therapeutic approach. *Clostridium butyricum* (*C. butyricum*) plays a pivotal role in shaping microbial composition, preserving intestinal barrier integrity, and enhancing mucosal immunity. Its major metabolites, short-chain fatty acids (SCFAs), further strengthen mucosal defenses and exert antiviral and anti-inflammatory effects. This review proposes a unified “gut-centric hypothesis” that intestinal barrier integrity, microbial homeostasis, and mucosal immune balance collectively determine the host’s resilience to viral invasion and inflammation. The collective findings delineate a mechanistic axis whereby *C. butyricum* orchestrates antiviral and anti-inflammatory immunity through the induction of type I/III interferons, modulation of inflammasome signaling, and expansion of regulatory immune populations, reinforcing its therapeutic promise. This review provides a new conceptual framework linking probiotic action to antiviral immunity, identifying *C. butyricum* as a potential next-generation microbial therapeutic for viral and inflammatory diseases.

## Graphical Abstract

**Figure:**
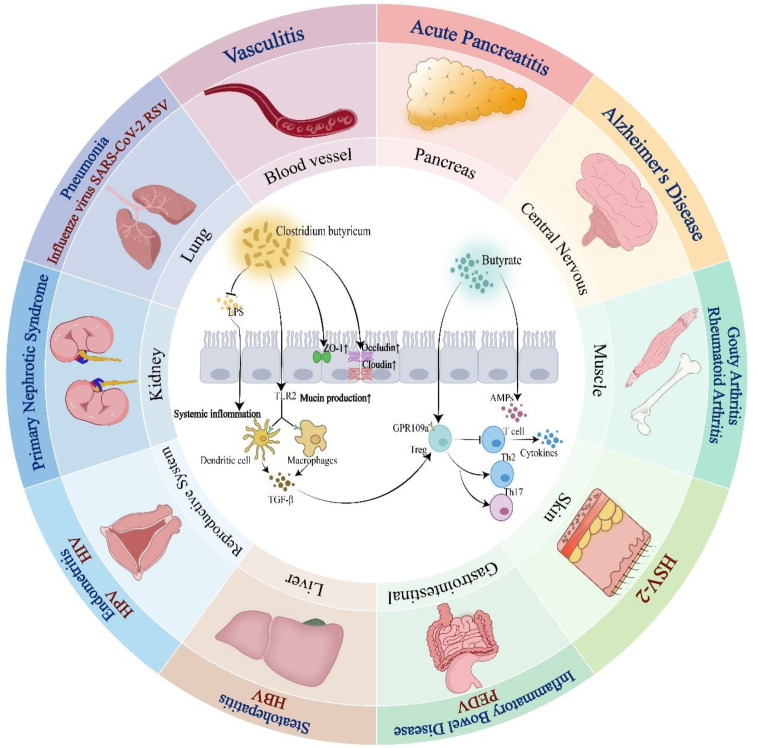

## 1 Introduction

*Clostridium butyricum*, an anaerobic bacterium that produces butyrate and forms spores, is vital for gut health. Prazmowski first isolated it from the intestinal tract of pigs in 1880 due to its unique metabolic properties (1). *C. butyricum* produces short-chain fatty acids (SCFAs) via the butyrate kinase (buk) pathway, which are primary metabolites formed by the bacterial fermentation of dietary fiber in the gut (1, 2). These metabolites protect intestinal health and promote bile acid metabolism, influencing the host’s health status (3). As a key probiotic, *C. butyricum* is widely used in Japan, South Korea, China, and other regions. It plays a crucial role in balancing gut microbiota, enhancing immunity, and preventing gut-related diseases (1).

Recent interest has grown in the potential of *C. butyricum* and SCFAs in combating viruses and alleviating inflammation. The gut, as the body’s largest immune organ, maintains mucosal immune homeostasis, defends against external threats, and oversees self-surveillance. *C. butyricum* modulates the activity of mucosal immune cells and inflammatory factors, alleviating inflammatory responses and enhancing defense against viral infections. It strengthens the body’s ability to defend against pathogens and fosters immune tolerance. Additionally, *C. butyricum* directly combats viral infections by inhibiting viral invasion and replication. SCFAs primarily exert effects through gene and protein regulation, suppressing histone deacetylases to modulate virus-related gene expression and enhance interferon production. By activating G-protein-coupled receptors, they refine the functions of intestinal dendritic cells, macrophages, and other immune cells, amplifying both antiviral and anti-inflammatory responses, thus playing a pivotal role in mucosal immunity.

This review proposes the “gut-centric hypothesis”, highlighting the role of *C. butyricum* in regulating gut immunity. *C. butyricum* is a promising drug target that enhances intestinal barrier function and modulates type I/III interferon responses, providing strategies against emerging viruses. Its dual activity in antiviral and anti-inflammatory responses underscores its potential in clinical treatments. This research offers a new perspective for foundational studies in mucosal immunology and has broad applications in developing interventions for mucosal immunity, addressing viral infections, and treating inflammatory diseases.

While *C. butyricum*’s role in gut immunity has been noted, several areas require further exploration. This review addresses critical gaps: the mechanisms of *C. butyricum* in gut mucosal immunity, its specific applications in viral infections and inflammatory diseases, and its potential as an antiviral and anti-inflammatory therapeutic bacterium.

## 2 Antiviral functions and inflammation alleviation of *C. butyricum* and its key metabolites

*C. butyricum* is known for its production of SCFAs and its ability to adjust host immune and metabolic functions. This review explores how *C. butyricum* and its SCFAs induce intestinal mucosal immunity in antiviral defense and alleviate inflammation, focusing on their effects on gut microbiota, barrier function, immune regulation, inflammatory signaling pathways, and antiviral responses.

### 2.1 *C. butyricum* and its key metabolites regulate gut microbiome

As a probiotic, *C. butyricum*’s primary function is to regulate intestinal health. It modulates intestinal mucosal immunity by influencing gut microbiota and enhancing its defensive function. It promotes the growth of beneficial bacteria, especially those with high butyryl-CoA gene content, and inhibits pathogenic species proliferation (4). SCFAs lower the pH in the intestinal lumen, inhibit harmful bacteria proliferation, and optimize microbiome structure (4). The increase in Lactobacillus and Bifidobacterium was observed in mouse models supplemented with *C. butyricum*, further validating their role in regulating the gut ecosystem. *C. butyricum* regulates bile acid levels to inhibit the proliferation and cytotoxin production of *Clostridioides difficile*. It also competes with *Helicobacter pylori* for adhesion sites on gastric epithelial cells, effectively suppressing its growth (5, 6). Furthermore, *C. butyricum* significantly inhibits multiple pathogenic bacteria, including *Staphylococcus aureus*, *Vibrio cholerae*, *Shigella flexneri*, and *Salmonella* spp, by secreting antimicrobial peptides (7–10). It also prevents infections caused by enterohemorrhagic Escherichia coli (7).

On a molecular level, *C. butyricum* modulated gut microbiota structure by inhibiting Wnt/β-catenin pathways (9). This resulted in two primary outcomes: inhibition of pathogenic bacteria and bile acid-metabolizing strains, and increased proliferation of SCFA-producing microbial communities.

### 2.2 *C. butyricum* and its key metabolites modulate the intestinal barrier

The intestinal barrier is essential for mucosal immunity. When the barrier is compromised, bacteria and toxins can enter the bloodstream, triggering autoimmune and inflammatory responses. *C. butyricum* and its metabolites enhance intestinal barrier function, preventing pathogens from penetrating the intestinal epithelium and strengthening mucosal immune defense. Butyric acid, the primary energy source for intestinal epithelial cells, promotes metabolism and repair, enhances stress tolerance, and stabilizes barrier function (1). SCFAs upregulated tight junction proteins such as ZO-1, occludin, and cadherin by inducing local hypoxia and activating hypoxia-inducible factor, reinforcing intercellular junctions (11, 12). Additionally, SCFAs improved the transmembrane resistance of epithelial cells by activating the AMPK pathway and reducing apoptosis via the PI3K/Akt signaling pathway (13). Butyrate also stimulated mucin secretion in goblet cells and activate the MAPK pathway, contributing to improved infection resistance (14).

### 2.3 Gut-immune axis: *C. butyricum* and its key metabolites regulate immune cell differentiation and function

The gut-immune axis connects gut microbiota, the intestinal immune system, and systemic immune responses. C. butyricum influences the intestinal mucosal immune response against viruses and inflammation by modulating T cells, B cells, and dendritic cells function and differentiation. It promotes regulatory T cells differentiation, and modulates T and B cell function. Administration of C. butyricum in mouse models significantly enhanced Treg differentiation and abundance. SCFAs promoted Treg differentiation and stimulated IL-22 production by CD4^+^T cells via G protein-coupled receptor 41 and HDAC function, boosting immune modulation (15, 16). These findings indicate that SCFAs contribute to anti-infection immunity by influencing T-cell differentiation. SCFAs also directly modulated T-cell differentiation and participated in cell-specific immunity. Butyrate did not inhibit FoxP3^+^ T cells but impeded the proliferation of CD4^+^ T cells, likely due to its immunomodulatory specificity or ability to regulate genes associated with lymphocyte differentiation (17). Additionally, butyrate enhanced immunomodulation by combining GPR43-induced granzyme B (GZMB) (18).

*C. butyricum* significantly impacts B-cell maturation. SCFAs promote metabolic pathways, including acetyl-coenzyme A biosynthesis, oxidative phosphorylation, glycolysis, and fatty acid biosynthesis (19). As an HDAC inhibitor, SCFAs upregulate the genes related to B-cell differentiation, such as *Aicda* and *Prdm1*, thereby supporting B-cell differentiation and maturation (19). This reduces circulating IgE levels, alleviating allergic reactions while supporting antibody production and adaptive immune responses (20). SCFAs also regulate the intestinal mucosal immune response by modulating the differentiation, maturation, and activation of various immune cells, including dendritic cells, macrophages, and T cells, through the TLR2 pathway. By inhibiting HDAC activity, SCFAs induce the production of B10 cells with anti-inflammatory functions. These mechanisms are crucial for maintaining intestinal mucosal immune homeostasis and combating inflammation.

### 2.4 *C. butyricum* and its key metabolites modulate inflammation-related pathways

Inflammatory factors play a critical role in intestinal mucosal immunity. C. butyricum modulates the intestinal immune response by regulating inflammatory factor expression, preventing excessive inflammation, and protecting the gut from pathogen invasion. *C. butyricum* and SCFAs regulate inflammatory factors through the NF-κB and TLR4 signaling pathways. The inflammatory process is key in the onset and progression of many pathological conditions. Activating TLR4 enhances anti-inflammatory cytokines generation, such as IL-10, while inhibiting pro-inflammatory mediators like IL-1β and IL-6. *C. butyricum* facilitates retinol metabolism, increasing retinoic acid levels to further alleviate inflammatory responses (21). By promoting prostaglandin E2 (PGE2) synthesis and inhibiting matrix metalloproteinase-9 (MMP-9) expression, SCFAs improve infection-induced immunopathological states (22). Inhibiting mast cell degranulation also alleviates respiratory inflammation and reduces tissue damage.

SCFAs significantly decrease the release of pro-inflammatory chemokines, including CCL3, CCL4, CCL5, CXCL9, CXCL10, and CXCL11, impairing immune cell migration to inflammation sites. Butyrate reduces LPS-induced pro-inflammatory cytokine expression by inhibiting key TLR4 pathway molecules, such as TRAF6, TRAF3, and IRF3 (23–26). Butyric acid also reduces NF-κB activation by downregulating TRAF6, further inhibiting pro-inflammatory cytokines transcription.

### 2.5 *C. butyricum* and its key metabolites interact with viruses and inflammatory processes

The mucosal immune system combats pathogens entering the body. *C. butyricum* and its metabolites activate intestinal mucosal immune responses, enhancing the gut’s defense against viruses while modulating inflammation. *C. butyricum* promotes interferon production, activates the NF-κB pathway to inhibit inflammatory signaling molecules, and replicates various RNA and DNA viruses (27, 28). Furthermore, *C. butyricum*’s antiviral and anti-inflammatory effects depend on the key components within its metabolites—SCFAs. These limit viral spread by regulating JAK and IRF pathways and inhibiting endothelial cell adhesion molecule expression, thereby enhancing host immune defense (29, 30). They also modulate intestinal immune cells to inhibit inflammation and maintain host immune homeostasis.

## 3 Antiviral mechanisms of *C. butyricum* and its key metabolites

### 3.1 Influenza virus

Influenza viruses are classified into four types based on nucleoproteins and matrix proteins: A, B, C, and D (31). The subtypes of Influenza A that primarily infect humans are H1N1 and H_3_N_2_, while the lineages of Influenza B viruses are Victoria and Yamagata. Recently, *C. butyricum* has gained attention as an agent against influenza viruses.

**Figure 1:**
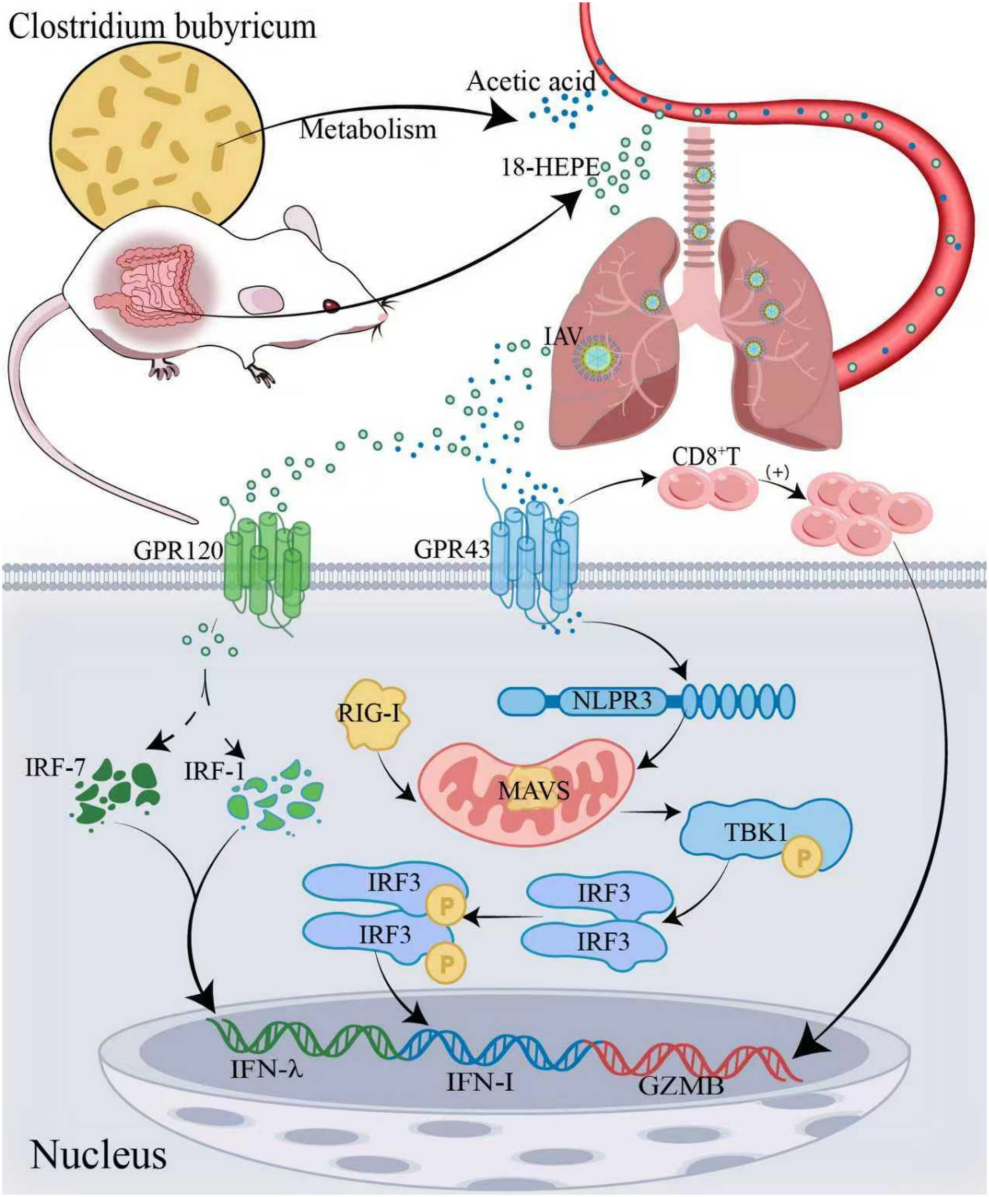
Mechanisms of *C. butyricum* against the influenza virus. Oral administration of *C. butyricum* in mice induces the production of ω-3 fatty acid 18-hydroxy eicosapentaenoic acid (18-HEPE), which activates GPR120 and IRF-1/-7, promoting the production of IFN-λ in lung epithelial cells and enhancing the ability to combat influenza. The metabolite acetate binds to GPR43, promoting CD8^+^ T cell generation and stimulating GZMB secretion. Through binding with the GPR43 receptor and in the presence of NLRP3, acetate enhances the virus RNA-triggered MAVS aggregation, promoting IRF3 activation and IFN-I production, thereby inhibiting the influenza virus spread.18-HEPE: 18-hydroxy-eicosapentaenoic acid; GPR120, G protein-coupled receptor 120; IRF-1/-7, Interferon regulatory factors 1/-7; IFN-λ, Interferon-λ; GPR43, G protein-coupled receptor 43; CD8^+^ T cells, CD8^+^ T lymphocytes; GZMB, Granzyme B; NLRP3, NOD-like receptor family pyrin domain-containing protein 3; MAVS, Mitochondrial antiviral-signaling protein; IRF3, Interferon regulatory factor 3; IFN-I, Type I interferons.

Oral administration of *C. butyricum* alleviated inflammation caused by the influenza virus in a murine model, potentially due to FFAR3 and fatty acid β-oxidation pathway stimulation that enhances CD8^+^ T cells activity (32). *C. butyricum* can directly modulate intestinal mucosal immunity, enhancing the antiviral activity of CD8^+^T cells by binding to GPR43 and inducing Acetyl-CoA production (33). These processes increase oxidative phosphorylation, glycolysis, and other metabolic functions. Interferons play a central role in the intestinal mucosal immune response. *C. butyricum* upregulates IFN-λ production by inducing the ω-3 fatty acid 18-hydroxy eicosapentaenoic acid (18-HEPE), which activates G protein-coupled receptor 120(GPR120) and interferon regulatory factors (IRF-1 and IRF-7) (34, 35). IFN-λ interacts with the IFNLR1 on the cell surface to activate the JAK-STAT pathway, initiating antiviral gene expression. The proteins encoded by these genes inhibit viral replication and prevent viral assembly and release. Additionally, IFN-λ inhibits macrophage infection and enhances the host immune response (36). The Acetate-GPR43-NLRP3-MAVS-IFN-I pathway was proposed as a potential target for treating respiratory viral infections (37). (**Figure 1**) Acetate salts can also elevate IFN-β levels, enhancing antiviral capabilities (38). The inhibitory effect of butyrate on the influenza virus was confirmed by a positive correlation between butyrate levels and lymphocyte ratio, as well as MxA(GPR120) expression, and negatively with viral load (39). Acetate also reduced influenza viral load by enhancing anti-inflammatory mediators.

### 3.2 SARS-CoV-2

SARS-CoV-2, a respiratory virus, also impacts the gastrointestinal tract. The metabolites of *C. butyricum* are crucial in combating SARS-CoV-2 infection. SARS-CoV-2 infection causes a leaky gut and systemic inflammation. Butyrate, an energy source for intestinal epithelial cells, restores intestinal barrier integrity by promoting tight junction proteins like Claudin-1 and Occludin. It enhances intestinal barrier function and boosts immune defense against viruses by promoting Mucin and antimicrobial peptide secretion from goblet cells (40). Clinical research confirms that calcium butyrate improves gut microbiota dysbiosis in SARS-CoV-2 patients, reduces secondary infections, and decreases virus-induced respiratory damage (41).

SARS-CoV-2 infections often involve a cytokine storm, releasing large amounts of pro-inflammatory cytokines and causing systemic inflammation (42). Butyrate impeded the release of pro-inflammatory cytokines by binding to the G protein-coupled receptor 109a(GPR109a), activating regulatory Tregs and inhibiting overactive T cell responses (16, 40, 43), thus regulating intestinal mucosal immunity and alleviating systemic inflammation.

It also activates M2-type macrophages, increasing the anti-inflammatory factor IL-10 and decreasing pro-inflammatory factor IL-6 levels. Additionally, propionate inhibits the overactivation of pro-inflammatory signaling pathways and HDAC activity, attenuating the cytokine-mediated systemic inflammatory response.

**Figure 2:**
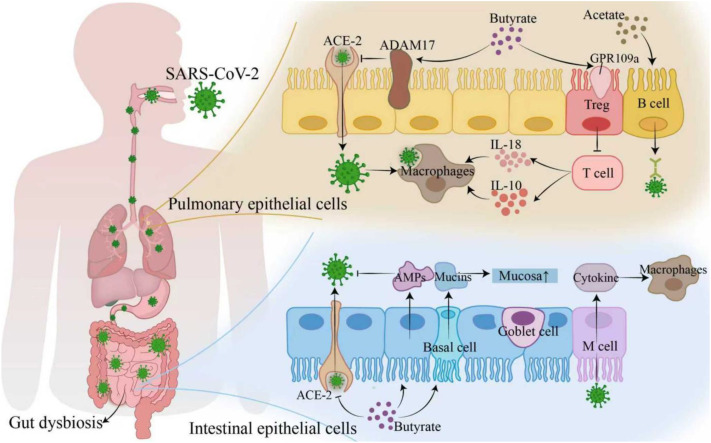
Mechanisms of *C. butyricum*-derived butyrate against SARS-CoV-2. In lung epithelial cells, butyrate upregulates t Adam17, inhibiting SARS-CoV-2 binding to the ACE-2 receptor and preventing viral entry. It also regulates Treg cells via the GPR109a receptor, suppressing excessive T-cell activation and reducing inflammatory factors IL-10 and IL-18. In intestinal epithelial cells, butyrate promotes mucus (Mucins) and antimicrobial peptides (AMPs) secretion by goblet cells, protecting the intestinal barrier. Adam17, A disintegrin and metalloproteinase 17; ACE-2, Angiotensin-converting enzyme 2; GPR109a, G protein-coupled receptor 109a; Treg, Regulatory T cells; IL-10, Interleukin-10; IL-18, Interleukin-18; Mucins, Mucins; AMPs, Antimicrobial peptides.

Butyrate and acetate inhibit viral replication and enhance host immunity through various mechanisms. Butyrate hinders SARS-CoV-2 binding to the angiotensin-converting enzyme 2 (ACE2) receptor by upregulating Adam17, which prevents the virus from entering host cells (43, 44), thereby enhancing the defensive function of the intestinal immune system. Besides, butyrate directly inhibited SARS-CoV-2 replication by downregulating HMGB1 expression (43). Additionally, it also upregulated the intracellular expression of IRF7 and interferon receptor through calmodulin phosphatase-binding protein-1 and the TLR signaling pathway, enhancing host resistance to the virus (45, 46). Acetate stimulated B cells to produce specific antibodies against SARS-CoV-2, further inhibiting virus transmission (**Figure 2**).

### 3.3 Human immunodeficiency virus

HIV is a global public health concern due to its immunoinhibition and destruction of CD4^+^T cells. Although antiretroviral therapy(ART) can inhibit viral load effectively, complete eradication of HIV remains challenging. *C. butyricum* has gained attention for its ability to modulate intestinal immunity and inflammatory response.

*C. butyricum* inhibits microbial translocation due to HIV infection by reducing harmful bacteria. It replenished lost CD4^+^ T cells after HIV infection, which enhanced intestinal immune function (47). SCFAs, which are the key metabolites, also play an important role in immune response. Specifically, propionate mitigated HIV-induced immune system destruction through Th1 and Th17 cell inhibition and facilitates regulatory Treg development, preserving immune homeostasis and reducing chronic inflammation. Acetate reduced inflammation by inhibiting neutrophil migration (48). Additionally, *C. butyricum* induces the production of type I interferon and increases the expression of antiviral proteins. For example, APOBEC family proteins and bone marrow stromal antigen 2 (BST-2) proteins inhibited HIV replication through gene mutation and inhibiting viral release. Type I interferon not only counteracted the HIV-mediated immune escape response but also enhanced the antiviral response of plasmacytoid dendritic cells (PDCs). This improves the immune response in HIV-infected patients.

However*, C. butyricum* also exerts a detrimental effect on HIV. Butyrate may accelerate the progression of HIV infection by inhibiting HDAC activity and promoting HIV-1 gene expression. Furthermore, acetate significantly decreased the release of α-defensin, an active anti-HIV molecule, in older women, weakening the immune system’s ability. Thus, the influence of *C. butyricum* on HIV is dual: it modulates intestinal immunity and induces interferon production, while potentially promoting viral progression under certain conditions.

### 3.4 Herpes simplex virus type 2

Herpes Simplex Virus Type 2 is a common pathogen that causes recurring diseases. As a beneficial gut bacterium, *C. butyricum* improves the skin and mucosa’s microenvironment, enhancing barrier integrity and immune function and protecting against viral invasion. It hampers HSV-2 replication *in vitro*. Lactic acid, a metabolite of *C. butyricum*, inhibits the envelope fusion glycoproteins of HSV-2 by altering environmental pH, thus preventing the virus’s entry and spread (49). It also strengthens the body’s ability to combat viral infections by releasing IFNs and pro-inflammatory cytokines (50). Additionally, *C. butyricum* regulates immune cell proportions by promoting regulatory Treg generation and reducing Th1 and Th17 activation, which inhibits HSV-2 spread and reduces tissue damage caused by infection (27).

### 3.5 Respiratory syncytial virus

**Figure 3:**
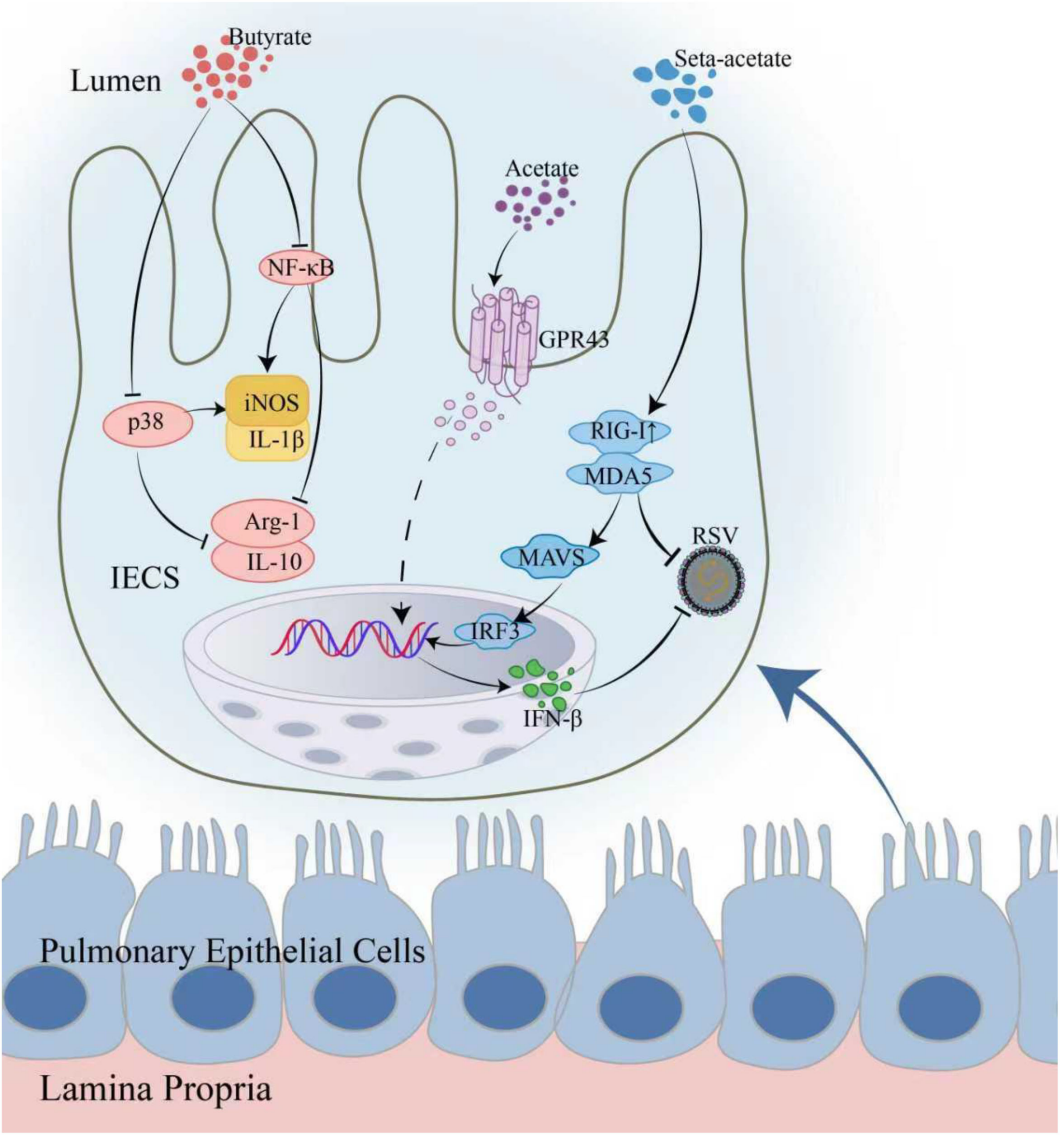
Mechanisms of *C. butyricum* metabolites against RSV infection. In lung epithelial cells, butyrate inhibits the NF-κB and p38 signaling pathways, downregulating pro-inflammatory factors iNOS and IL-1β, while upregulating the expression of anti-inflammatory factors Arg-1 and IL-10, effectively alleviating inflammation induced by RSV infection. Acetate activates the GPR43 receptor, inducing the production of IFN-β and enhancing the host’s antiviral capability. Additionally, acetate can directly inhibit viral replication by upregulating the expression of RIG-I, and by triggering MAVS aggregation, it induces the production of interferons to exert antiviral effects.NF-κB, Nuclear factor kappa B; p38, p38 signaling pathway; iNOS, Inducible nitric oxide synthase; IL-1β, Interleukin-1β; Arg-1, Arginase-1; IL-10, Interleukin-10; GPR43, G protein-coupled receptor 43; IFN-β, Interferon-β; RIG-I, Retinoic acid-inducible gene I protein; MAVS, Mitochondrial antiviral-signaling protein; IRF3, Interferon regulatory factor 3.

RSV is a seasonal virus that severely affects children under two years of age, leading to viral bronchiolitis. *C. butyricum* helps combat the virus through various mechanisms. It protects the gut barrier by reducing dysbiosis and improving gut function disrupted by RSV infection, thus indirectly alleviating lung inflammation. It reduced the severity of lung inflammation and decreased tissue damage by modulating the gut-lung axis. Studies showed that the risk of RSV infection is significantly reduced in allogeneic hematopoietic cell transplant recipients (allo-HCT) with higher levels of butyrate-producing bacteria (51). In animal experiments, butyrate enhanced anti-inflammatory and repair capabilities by reducing inflammatory cell infiltration in the lungs and promoting macrophage polarization toward the M2 phenotype (52). Acetate downregulated pro-inflammatory factors iNOS and IL-1β, and upregulated anti-inflammatory factors Arg-1 and IL-10. Propionate induces type-I interferon-β production and up-regulates antiviral ISGs by GPR43, thereby inhibiting RSV replication and spread. Acetate activated the GPR43 receptor to promote IFN-β production and strengthen host antiviral defenses in lung epithelial cells (53). It also enhanced host recognition of RSV RNA by upregulating RIG-I expression, inhibiting viral replication, and decreasing viral load (54) (**Figure 3**).

### 3.6 Hepatitis B virus

Approximately 296 million people are infected with HBV, a major cause of liver cirrhosis and hepatocellular carcinoma. Probiotic therapy has been recognized as an adjunctive therapy to treat liver diseases related to HBV, due to its effects on regulating gut flora and improving liver function. Recent studies show that *C. butyricum* can enhance liver function and alleviate symptoms in HBV-induced cirrhosis (55).

**Figure 4:**
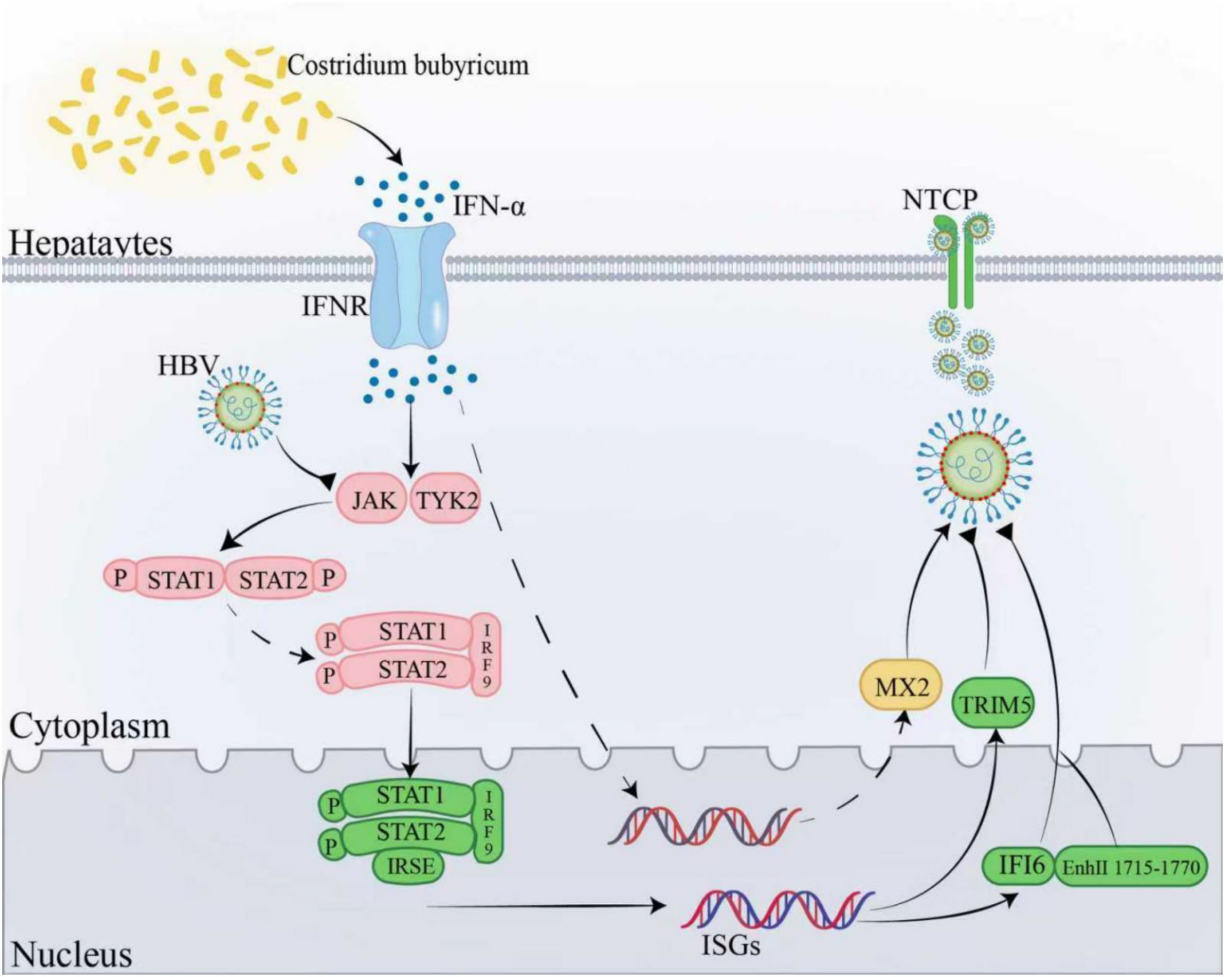
Mechanisms of *C. butyricum* against HBV infection. *C. butyricum* stimulates IFN-α production upon binding to its receptor and activates the JAK/STAT signaling pathway. This will trigger a cascade of events that induces the expression of ISGs and the synthesis of the antiviral protein TRIM5. Furthermore, *C. butyricum* can activate the IFI6 gene, which interacts with the EnhII/Cp promoter region (nt1715-1770), inhibiting HBV replication. However, under certain circumstances, interferon-induced MX2 may paradoxically facilitate HBV replication. IFN-α, Interferon-alpha; JAK/STAT pathway, Janus kinase/signal transducer and activator of transcription pathway; ISGs, Interferon-stimulated genes; TRIM5, Tripartite motif-containing protein 5; IFI6, Interferon-induced protein 6; EnhII/Cp promoter, Enhancer II/core promoter; MX2, Myxovirus resistance protein 2.

Sodium butyrate reduced LPS entry into the bloodstream and inhibited the TLR4-mediated increase in intestinal epithelial permeability, which preserved intestinal barrier integrity (56). Simultaneously, *C. butyricum* alleviated chronic liver inflammation and immune dysregulation caused by HBV infection by regulating inflammatory factors. Additionally, it induced the production of type I interferons, activating the expression of ISGs and the antiviral protein TRIM5 (57). After binding to its receptor, type I interferons activate the JAK/STAT signaling pathway, which in turn activates the IFI6 gene (58). The activated IFI6 can downregulate HBV gene expression and inhibit its replication. HBV infection is known to generate excessive superoxide radicals in host cells, leading to oxidative stress. However, extracellular polysaccharides of *C. butyricum* possess strong antioxidant properties, protecting DNA from damage (59). It is important to note that interferon-induced MX2 and IFIT3 promote HBV replication in some cases (60) (**Figure 4**). Current research is insufficient and lacks clinical trials; further investigation into the specific mechanisms of *C. butyricum* in HBV infection and its clinical efficacy is needed.

### 3.7 Other viruses

Previous sections discussed the antiviral activities of *C. butyricum* against specific viruses. It also exhibits antiviral activities against other viruses. The following discussion presents the antiviral activities of *C. butyricum* against additional viral infections, focusing on immune regulation and viral replication inhibition.

Sodium butyrate activates the GPR109a receptor and regulates the PERK-eIF2α signaling pathway, inhibiting ERS-mediated apoptosis (61). This mechanism is crucial in RV infection, protecting cells from virus-induced apoptosis and providing potential intervention points for RV prevention and control. *C. butyricum* modulates interferon-related factors to inhibit BVDV replication (62). It also inhibited PEDV replication through the NF-κB pathway, induced interferon production and downstream ISGs, thereby enhancing antiviral potency (63). Apart from that, *C. butyricum* inhibited the replication of NDV efficiently *in vitro (*27). *C. butyricum*’s antiviral action also involves immune regulation. SCFAs that *C. butyricum* produces activate the gut-liver axis immune pathway to regulate CD8 T-cell expression and enhance the host’s immune response against FAdV-4 (64). They also caused T lymphocytes and monocytes to adhere to endothelial cells to increase the immunological defenses of the body against EHV-1 (29). During the primary stage of CAHV infection, *C. butyricum* induced a strong immune response, enhancing Longfin tuna survival rates (29). Clinical studies demonstrated that *C. butyricum* significantly reduces the risk of lower respiratory tract viral infections in patients undergoing allo-HCT (51). *C. butyricum*, its key metabolic product SCFAs, and prebiotics exert distinct effects on various viruses in animal experiments. However, clinical trials are still lacking to confirm these findings in humans (**Table 1**).

**Table 1:** *C. butyricum* and its probiotics in antiviral animal models.

Probiotics, prebiotics, and SCFAs	Experimental subjects	Dosage of probiotics, prebiotics, and SCFAs	Virus type	Results	Reference
*C. butyricum*	Mice	500 mg/kg/day	Influenza virus	Mice supplemented with *C. butyricum* showed significantly lower mortality and viral load in the lungs compared to the control group. Additionally, IFN-λ expression was upregulated.	(32)
Acetate, Propionate, and Butyrate	1-day-old SPF Chickens	Sodium acetate (80 mM), Sodium propionate (10 mM), and Sodium butyrate (20 mM)	Fowl adenovirus-4 (FAdV-4)	Chickens supplemented with SCFAs showed significantly higher survival rates during acute viral infection compared to the control group. Additionally, the number of activated T cells and MHC II-expressing monocytes increased.	(64)
Butyrate	Mice	Sodium butyrate (200 mg/kg)	Bovine viral diarrhea virus (BVDV)	Butyrate treatment significantly reduced BVDV RNA levels in the duodenum, jejunum, spleen, and liver while significantly increasing the expression of ZO-1 mRNA and protein induced by BVDV.	(62)
*C. butyricum*	Gilthead seabream (~5 g average weight)	1 × 10^4^ cfu/g added to the diet and 1 × 10^6^ cfu/L added to water	Carp herpesvirus (CaHV)	The experimental group with *C. butyricum* had a significantly reduced viral load and increased survival rate compared to the control group. Moreover, expression levels of innate immunity-related genes (e.g., IL11, IRF7, PKR, and Mx) were also elevated.	(65)
Acetate, Propionate, and Butyrate	Pigs	500 μM acetate, propionate, and butyrate	Porcine Epidemic Diarrhea Virus (PEDV)	The supplementation with SCFAs significantly reduced viral load and upregulated the expression of IFN and ISGs.	(63)
*C. butyricum*	Mice	1 × 10^8^ CFU/mL, 200 μL/day	Respiratory Syncytial Virus (RSV)	The *C. butyricum*-supplemented group showed reduced total inflammatory cells and viral load in the lungs of RSV-infected mice compared to the control group. Additionally, levels of macrophages, lymphocytes, and neutrophils were decreased.	(52)
High-fiber diet + Acetate, Propionate, or Butyrate (one of them)	Mice	Final concentration 200 mM in drinking water	Respiratory Syncytial Virus (RSV)	The group supplemented with SCFAs had significantly lower viral loads and lung inflammation compared to the control group, with increased IFN-β levels.	(53)
Sodium butyrate (SB), Sodium propionate (SPr), and Sodium acetate (SAc)	Horses	0.5 or 5 mM SB, SPr, and SAc	Equine Herpesvirus 1(EHV1)	The group supplemented with short-chain fatty acids showed reduced innate immune responses in the upper respiratory tract post-EHV1 infection and decreased adhesion of blood-derived monocytes and T lymphocytes to horse endothelial cells.	(29)
Butyrate	Mice	20 mmol/L sodium butyrate	Human papillomavirus (HPV)	HPV16E6/E7 immortalized keratinocytes treated with butyrate had significantly extended survival times and improved cell differentiation compared to the control	(66)
Inulin (dietary fiber)	Mice	30% inulin supplementation	Influenza virus	The group supplemented with inulin showed reduced respiratory inflammation, vascular damage, and subsequent hemorrhage, with activated CD8 T-cells enhancing antiviral immunity compared to the control group.	(32)
Sodium butyrate, Sodium propionate, Sodium acetate mixture	Mice	67.5 mM Sodium butyrate (Sigma 303410), 40 mM Sodium acetate (Sigma S2889), and 25.9 mM Sodium propionate (Sigma P1880)	SARS-CoV-2	The mixture of butyrate, propionate, and acetate significantly reduced the expression of ACE2 in the gut and lungs and inhibited nasal infections. Additionally, it improved coagulopathy associated with SARS-CoV-2.	(67)

## 4 Alleviating inflammatory diseases: mechanisms of *C. butyricum* and its metabolites

### 4.1 Digestive system inflammatory diseases

Inflammatory bowel disease (IBD) involves inflammatory lesions in the rectum and colon. *C. butyricum* helps alleviate colitis. Mice treated with *C. butyricum* showed reduced inflammatory cell infiltration and less damage to the mucus layer in the colon compared to the control group. It also significantly decreased intestinal pathogens like Shigella and Escherichia coli (68). High concentrations of butyrate (80–140 mM) reduced epithelial cell proliferation in colitis mice and enhanced tissue repair (69). Additionally, *C. butyricum* inhibited TLR2 signaling and IL-17 secretion, improving the local mucosal immune response and providing protection against intestinal inflammation.

*C. butyricum* shows potential therapeutic benefits of in liver inflammation, particularly in hepatitis and steatohepatitis. Gut-liver immune modulation has emerged as a promising approach in probiotic therapy for liver diseases. In a mouse model of steatohepatitis, the treatment with *C. butyricum* resulted in a significant reduction in inflammatory responses (70). The levels of inflammatory factors decreased significantly, while the butyrate content in the gut increased markedly. This result was confirmed in clinical trials with NAFLD patients (71). Additionally, *C. butyricum* alleviates gut microbiota imbalance in NAFLD patients, reduces blood lipid levels, alleviates liver fibrosis, and mitigates liver function damage.

Acute pancreatitis (AP) is a common abdominal inflammatory disease. In mouse models, *C. butyricum*can prevent AP, possibly by inhibiting neutrophil and dendritic cell infiltration in the pancreas. It also inhibits the TLR4 signaling pathway and the formation of the NLRP3 inflammasome (72). Gut microbiota analysis in mice confirmed *C. butyricum*’s beneficial effects on gut-pancreas axis homeostasis, showing a significant reduction in *Desulfovibrionaceae* and increased abundances of *Verrucomicrobiaceae*, *Clostridiaceae*, and *Lactobacillaceae*.

### 4.2 Respiratory system inflammatory diseases

Upper respiratory tract inflammation, exemplified by eosinophil-dominated chronic sinusitis, is characterized by as type 2 inflammation with an increase in eosinophils, as well as elevated production of type 2 cytokines such as IL-5 and IL-13. Butyrate inhibits the production of type 2 cytokines in nasal polyp-derived cells (73). This may be achieved by inhibiting IL-6 and TNF-α production, leading to macrophage polarization toward the M2 type. In the future, *C. butyricum* may serve as a potential therapeutic target for ECRS.

For lower respiratory tract inflammation, such as allergic bronchitis, and pneumonia. *C. butyricum* treatment significantly alleviates allergic airway inflammation and mucus secretion in allergic mice (74). Among the administration routes, aerosol delivery is the most effective, potentially enhancing T cell differentiation and inhibiting NLRP3 inflammasome signaling pathways.

### 4.3 Urogenital system inflammatory diseases

Chronic endometritis is linked to an imbalance in the female reproductive tract microbiota and pathogenic infections. Staphylococcus aureus is a common pathogen, and *C. butyricum* can inhibit its growth, thereby reducing tissue damage and inflammatory responses (75). Primary nephrotic syndrome (PNS) is a prevalent glomerular disease in children. The *C. butyricum* treatment group showed significant improvements in body weight and inflammatory responses. Butyrate, a metabolite of *C. butyricum*, is crucial for Treg cell differentiation. The balance between Th17 and Tregs is key in managing PNS-induced inflammation, suggesting that *C. butyricum* may mitigate the immune-inflammatory response in PNS by regulating the Th17/Tregs balance (76).

### 4.4 Musculoskeletal system inflammatory diseases

Rheumatoid arthritis, osteoarthritis, and gouty arthritis are common forms of arthritis characterized by bone destruction and joint degeneration. In mouse models, *C. butyricum* alleviated RA, potentially by producing butyrate to reduce IL-17-producing cells or by inhibiting HDAC2 in osteoclasts and HDAC8 in T cells, thus decreasing bone destruction (77, 78). *C. butyricum* reduced ACLT-induced bone destruction and loss (79). Additionally, it can inhibit the production of IL-1β, TNF-α, and cartilage-degrading matrix metalloproteinase-3 (MMP-3), blocking cartilage degradation, which may help in OA prevention and treatment. We observed that macrophage polarization levels correlated with the severity of gout (80). *C. butyricum* regulates macrophage polarization to counteract gouty arthritis by inhibiting miR-146a expression.

### 4.5 Circulatory and systemic inflammatory diseases

Vasculitis is a group of autoimmune diseases characterized by inflammation and necrosis of blood vessel walls. In a diabetic mouse model, supplementation with *C. butyricum* increases the level of its metabolite butyrate, activates the Nrf2 signaling pathway, and upregulates the HO-1 pathway, thereby reducing oxidative stress and alleviating HG-induced vascular inflammation (81). In studies of sepsis, *C. butyricum* stimulates intestinal epithelial cell proliferation and improves intestinal tissue damage. Intravenous administration of butyrate markedly reduces HMGB1 mRNA levels in rat tissues, alleviating the inflammatory response and protecting septic organs.

### 4.6 Neuroinflammatory diseases

**Figure 5:**
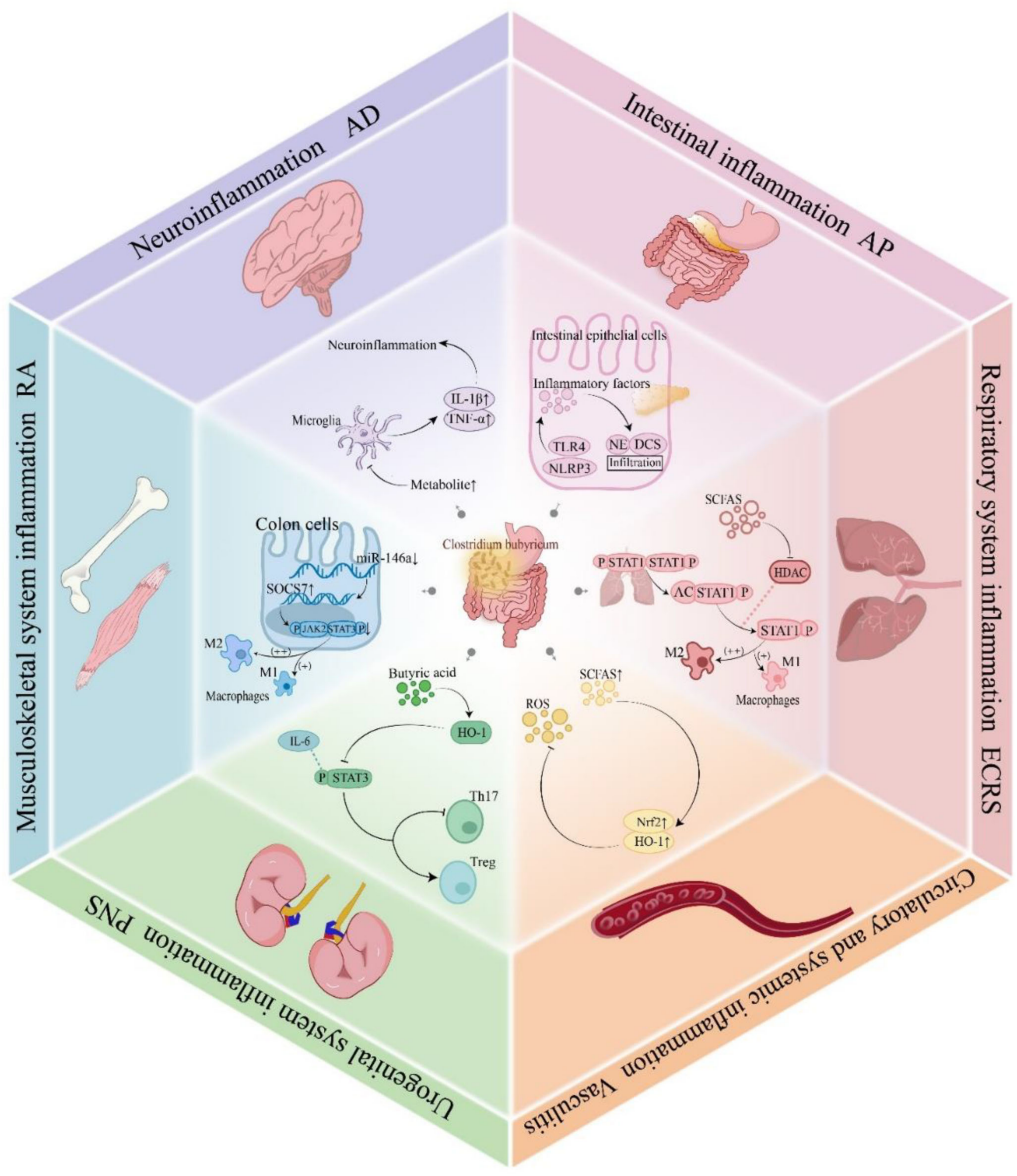
Mechanisms of *C. butyricum* in inflammatory diseases. *C. butyricum* colonizes the intestine and produces short-chain fatty acids that inhibit phosphorylation of the STAT1 signaling pathway, activate the Nrf2/HO-1 antioxidant pathway, downregulating the expression of miR-146a, prohibiting the overactivation of microglia, reducing inflammatory cell infiltration, and promoting M2 polarization of macrophages. These mechanisms provide regulatory effects on inflammatory diseases across six major systems, including the respiratory, circulatory, urogenital, musculoskeletal, neural, and digestive. STAT1, Signal Transducer and Activator of Transcription 1; Nrf2, Nuclear factor erythroid 2-related factor 2; HO-1, Heme oxygenase-1; miR-146a, MicroRNA-146a; IFN-α, Interferon-alpha.

Experimental autoimmune encephalomyelitis (EAE) is a chronic central nervous system autoimmune disease. Administration of *C. butyricum* significantly reduced neuropathological inflammation in the lumbar spinal cord. Compared to the control group, lymphocyte infiltration and myelin damage were both reduced (82). Furthermore, the incidence of physical disability was significantly lower in the *C. butyricum* treatment group (83). Similarly, in an Alzheimer’s disease (AD) mouse model, treatment with *C. butyricum* inhibited the phosphorylation of NF-κB p65 in Aβ-induced BV2 microglial cells, thus alleviating microglia-mediated neuroinflammation (**Figure 5**). *C. butyricum* produces distinct effects in different models of inflammatory diseases (**Table 2**).

**Table 2:** C*. butyricum* and its metabolites in inflammatory disease animal models.

Probiotic, prebiotic, and SCFA types	Experimental subjects	Dosage of probiotic, prebiotic, and SCFAs	Type of inflammation	Results	Reference
Butyrate	Mice infected with *Lactobacillus* in rodents	Butyrate (80–140 mM)	Colitis	Mice supplemented with butyrate showed a significant increase in body weight compared to the control group, a reduction in intestinal epithelial cell proliferation, and an increase in the expression of the Muc2 gene in the gut.	(69)
Synbiotic containing *C. butyricum* and Chitosan Oligosaccharides (COS)	Mice	10 CFU of Butyrivibrio fibrisolvens and 70 mg/kg COSs daily	Inflammatory Bowel Disease (IBD)	The experimental group showed significantly enhanced colon length, reduced inflammatory markers, and increased expression of tight junction proteins compared to the control group. Additionally, it reduced ROS levels and increased SCFA levels.	(84)
*C. butyricum*	Mice	200 μL Butyrivibrio fibrisolvens for 6 weeks	Primary Nephrotic Syndrome (PNS)	The treatment group showed improvements in weight loss, a decrease in 24-hour urinary protein levels, and correction of renal dysfunction in PNS mice.	(76)
*C. butyricum*	Mice	Supplemented with 10^8^ CFU/mL of Butyrivibrio fibrisolvens for 20 days	Colitis	Compared to the control group, the experimental group showed a significant reduction in inflammatory responses. IL-6 and IL-1β mRNA expressions were significantly elevated, while MUC2 expression was reduced. The cell composition was also altered, with a decrease in macrophages, dendritic cells (DCs), and mast cells.	(85)
*C. butyricum*	Mice	1 × 10^7^ CFU/200 μL PBS once daily for 7 days	Endometritis	After administration, there was a significant reduction in erythema induced by *Staphylococcus aureus*, and a marked reduction in MPO activity in the mice.	(75)
Clostridium butyric acid live bacterial capsules	NAFLD patients	400 mg Butyrivibrio fibrisolvens live capsule orally three times daily in addition to oral Rosuvastatin for 6 months	Hepatitis	The combined treatment group showed significant reductions in total cholesterol (TC), triglycerides (TG), free fatty acids (FFA), total bilirubin (TBIL), and direct bilirubin (DBIL).	(71)
*C. butyricum*	Mice	200 μL CB suspension every other day for 60 days	Vasculitis	Compared to the control group, the treatment group showed a significant increase in butyrate levels in blood vessels, a reduction in ROS levels, and an increase in Nrf2 and HO-1 levels.	(81)
*C. butyricum*	Mice	9.6 × 10^8^ CFU/kg/day	Pancreatitis	Compared to the control group, *C. butyricum* treatment alleviated AP-related intestinal inflammation and barrier dysfunction, inhibiting pathogen infiltration into the pancreas. The relative abundance of *Desulfovibrionaceae* decreased, while the abundance of *Clostridiaceae*, *Lachnospiraceae*, and *Lactobacillaceae* increased.	(72)
Sodium Butyrate	Mice	500 mg/kg/day via oral gavage	Rheumatoid Arthritis (RA)	Compared to the control group, the intervention group showed significantly higher levels of butyrate in the blood and altered macrophage polarization, with a reduction in M1 phenotype and an increase in M2 macrophages.	(80)
*C. butyricum*	Mice	0.2 mL daily by oral gavage	Allergic Airway Inflammation	The treatment group showed a significant reduction in airway inflammatory cell abundance and eosinophil recruitment, improving autophagy in lung cells of allergic mice.	(74)

## 5 Discussion

*C. butyricum* has significant potential in combating viruses and inflammation by regulating intestinal mucosal immunity. It induces interferon production, inhibits viral invasion and replication, and regulates inflammatory factors, demonstrating a powerful anti-infective effect. These mechanisms suggest that *C. butyricum* influences systemic immune responses through gut microbiota modulation, supporting the gut-immune axis and highlighting the gut’s role in overall health and disease.

We propose the “gut-centric hypothesis,” suggesting that the integrity of the intestinal barrier, gut microbiota balance, and optimal mucosal immune status are key components in the body’s response to viral invasion and inflammation mitigation. This serves as the basis for targeting probiotics in treating viral and inflammatory infections via the gut.

The antiviral impacts of *C. butyricum* and SCFAs depend on viral type and environmental conditions. For instance, butyrate modulates the immune response to HIV but can also increase HIV-1 and BZLF1 gene expression, activate latent viruses, and accelerate disease progression by inhibiting HDAC activity (86). Additionally, SCFAs inhibit the CXCR2 receptor, preventing CD4^+^T cell accumulation at infection sites and weakening host immunity (87, 88). High concentrations of SCFAs impair neutrophil responses to HIV by reducing the secretion of antimicrobial peptides, α-defensins, and chemokines, delaying the release of neutrophil extracellular traps (NETs) (48).

SCFAs have a dual role in regulating inflammation. They reduce the production of monocyte chemoattractant protein-1 (MCP-1) and LPS-induced IL-10 in human monocytes without affecting other cytokine and chemokine secretion, exerting an anti-inflammatory effect (89, 90). Conversely, they activate the NLRP3 inflammasome, promoting a pro-inflammatory response in inflammatory contexts. This dual effect may relate to differences in cell types and pathological environments (91).

Not all *C. butyricum* species are beneficial bacteria. Pathogenic *C. butyricum* strains include those producing botulinum neurotoxin E (BoNT/E), associated with infant botulism and adult intestinal botulism, and the minority strain MIYAIRI 588, which causes bacteremia in patients, a phenomenon potentially linked to genomic mutations (92–94).Some *C. butyricum* strains are associated with neonatal necrotizing enterocolitis (CB1002) (95).

## 6 Conclusions and future perspectives

This review highlights the potential applications of *C. butyricum* and its metabolites in fighting viruses and reducing inflammation. *C. butyricum* induces interferon production and significantly inhibits viral invasion and inflammation, showing promise as a clinical probiotic for enhancing intestinal immunity against infections.

There is substantial evidence supporting the use of *C. butyricum* in treating viral infections and inflammation; however, its mechanisms of action and role in modulating the gut-immune axis need further investigation. Probiotic therapy can restore a healthy mucosal immune status in the gut, facilitating effective responses to viral invasion and inflammation. Future research should focus on large-scale clinical trials to evaluate the efficacy and safety of *C. butyricum* in various viral infections, particularly gut and respiratory infections. Additionally, further investigation into the regulatory mechanisms of *C. butyricum* on the host immune system is necessary, especially regarding how different doses and ratios impact the intestinal barrier and immune status. Exploring combinations of *C. butyricum* with existing antiviral and anti-inflammatory drugs may optimize treatment strategies.

Currently, the application of *C. butyricum* faces several challenges, such as limited viral specificity and a y narrow antimicrobial spectrum. Clinical dosing is difficult to control, and it should be inactivated when used in combination with antibiotics. Efficacy varies among individuals, storage conditions are demanding, it is prone to inactivation at room temperature, and large-scale clinical trials are lacking. However, these limitations present opportunities for future research. By exploring these areas, we can advance the clinical application of *C. butyricum* in antiviral therapy and enhance our understanding of its regulatory mechanisms in intestinal immunity, supporting the development of effective and safe probiotic therapies for viral prevention and inflammation modulation.
